# Recasting Jung Through an Indigenist Approach to Deepen Shared Knowledges of Well-being and Healing on Australian Soils: Protocol for a Qualitative Landscape Research Study

**DOI:** 10.2196/36328

**Published:** 2022-12-08

**Authors:** Sophie Zaccone, Graham Jamieson, Clara V Murray, Mark J Lock, David Doyle

**Affiliations:** 1 School of Psychology Faculty of Medicine and Health University of New England Armidale Australia; 2 School of Health & Social Development Faculty of Health Deakin University Melbourne Australia; 3 Royal Flying Doctor Service Broken Hill Australia

**Keywords:** Jungian psychology, participatory research, participatory action research, co-design, marginalized, Indigenous, landscape research, qualitative research, qualitative methodology, user need, community need, relational study, ritual, Indigenist approach, First Nation, shared knowledge, knowledge sharing, well-being, healing, Australia

## Abstract

**Background:**

The colonization of Australia is responsible for complex layers of trauma for the First Nations peoples of the continent. First Nations Australians’ well-being is irrevocably tied to the well-being of the land. The application of a landscape-based approach to collaborative research shows promise in enabling genuine relationships that yield rich and informative data. However, there is a lack of practical evidence in the field of landscape research—research tied to First Nations Australians’ worldviews of landscape.

**Objective:**

This study aims to deepen shared knowledges of well-being and healing on Australian soils. We aim to examine ritual co-design as a novel method for deepening these shared knowledges.

**Methods:**

This research comprises a qualitative and participatory action research design operationalized through an Indigenist approach. It is a 2-phase project that is co-designed with First Nations Australians. Phase 1 of this project is a relational study that endeavors to deepen the theory underpinning the project, alongside the development of meaningful and reciprocal community connections. Phase 2 is a series of 3 participatory action research cycles to co-design a new communal ritual. This process seeks to privilege First Nations Australians’ voices and ways of knowing, which are themselves communal, ritual, and symbolic. The framework developed by psychiatrist Carl Jung informs the psychological nature of the enquiry. An Indigenist approach to landscape research recasts the Jungian frame to enable a culturally safe, context-specific, and landscape-based method of qualitative research.

**Results:**

The research is in the preliminary stages of participant recruitment. It is expected that data collection will commence in late 2022.

**Conclusions:**

It is expected that this qualitative and co-designed project will strengthen the cross-cultural co-designer relationships and that the data gathered from these relationships, and the accompanying practical outcomes, will provide new insight into the interaction between human and landscape well-being. The field of landscape research is in an embryonic phase. This new field is embedded in the understanding that First Nations Australians’ well-being is irrevocably tied to the well-being of the land, and this study seeks to build on this evidence base. A strength of this research is the relational methodology, in which First Nations Peoples’ needs and desires will inform future research directions. It is limited by its context specific nature; however, it is expected that findings will be usable in guiding future research directions in the multidisciplinary field of landscape research.

**International Registered Report Identifier (IRRID):**

PRR1-10.2196/36328

## Introduction

### Resisting Colonial Australia

We acknowledge the Elders, families and forebears of the Aboriginal and Torres Strait Islander peoples of the Australian continent, islands and adjacent seas, who remain the spiritual and cultural custodians of their lands and waters and who continue to practice their values, languages, beliefs and customs. [1]

This research acknowledges colonization as the genesis for complex layers of trauma in Australia, and it resists the colonial value system through the development of a culturally safe, empowering, and reflective mode of enquiry. It acknowledges that Australia’s history of colonization has seen the dispossession and dislocation of the continent’s “First People” from their lands and cultures though physical violence and political policies such as assimilation. This has resulted in complex layers of trauma for First Nations Australians [2].

Numerous reports with statistics of disadvantage in health and well-being [3]—known as deficit discourse [4]—indicate the gaps between First Nations (we respectfully use First Nations Australians to signify Aboriginal and Torres Strait Islander Peoples) and non–First Nations Australians. Underpinning the gaps is a history of dispossession, dislocation, and removal of First Nations children from their lands and families, resulting in the “Stolen Generation” [5]. Similar to other colonized parts of the world, this pattern reveals a long-lasting impact on the health and well-being of First Nations peoples and that colonization is an ongoing traumatic experience rather than a one-off historical event [6].

### Centering the Landscape in Research

For First Nation Australians, landscape or Country is a physical setting for performance and ritual but also the essential factor from which all other understandings of the universe stem, as Yolngu academic Dr Elaine Maypilama explains, “Country is land, air, water and stories of Dreaming” [7]. This landscape-based understanding of the world is dynamic and multilayered. It forms the “rules, norms and beliefs of existence between species and humans through connecting Aboriginal peoples’ back to ancestral beings from the time of creation” [8]. Mary Graham [9], a Kombumerri person, says, “The land is law. Land is a sacred identity and how we treat it determines our humanness...all meaning comes from land,” suggesting that the health of the land determines the health of First Nations people and culture, and the denial of these connections causes “unspeakable loss...and deep injury and trauma” [10]. For example, the recent Juukan Gorge destruction leaves both a physical wound to the land and a psychological wound on the souls of First Nations Australians, especially for Puutu Kunti Kurrama and Pinikura peoples [11].

### Healing Trauma Through Arts-Based Approaches

According to Bard and Yjindjarbandi researcher Dr Dawn Bessarab [12], First Nations communities use storytelling, performance, and visual arts to express culture—the cosmology and the interconnectedness of people, places, and histories [12]. Stories and myths are then enacted, shared, and presented in performances and rituals that provide a setting through which community members experience the most complete metaphorical expression of their cosmology [13]. Using arts-based approaches, which involve opportunities for storytelling and counter-storytelling, provides a valuable basis for developing meaningful healing and transformative research with First Nations peoples [14-16].

### Recasting Jung Through an Indigenist Approach

An Indigenist approach to the research takes Western theories and recasts them through methodological reform—where theories are challenged rather than imposed as given truth [17]. The research team comprising First Nations (MJL and DD) and non–First Nations (SZ, GJ, and CM) peoples proposes the Jungian framework as a novel approach to deepening the growing body of relational research in Australia. The Jungian framework is used for developing connections and research concepts between First Nations and non–First Nations Australians [18] through Jung’s understanding of the importance of meaning-making, spirituality, storytelling, and symbolism to human psychological well-being [19].

Jung’s search for understanding the essential connection between human and nature naturally resonates with First Nations cultures [20], who experience a kinship with Earth, grounded in systems of relationships and reciprocities that form the basis of all life [21]. Jung’s extensive study of comparative mythology and anthropology extended to First Nations Australians [22]. Petchkovsky [23] asserts that Jung’s concept of Active Imagination is a valid mode to understanding First Nations Australians’ land-based creation stories, performed rituals, and rites of passage that constitute human life and express cosmology.

### Aim

The aim of this study is to deepen shared knowledges of well-being and healing on Australian soils. The relational study endeavors to deepen the theory underpinning the project alongside the development of meaningful and reciprocal connections with First Nations Australians. From within these new connections, community needs and desires for research directions will be developed collaboratively. The objective of the research is to co-design a new communal ritual. Ritual co-design serves as both a research method and as a physical metaphor expressing the shared knowledges gained. The ritual co-design process explores First Nations Australians’ ways of knowing [24], which are themselves communal, ritual, and symbolic. Furthermore, feminist theory, women, Jung’s archetypal female principal, women’s practices, and ways of knowing have an essential and central role in this process.

## Methods

The methodology is a qualitative and participatory action research (PAR) design operationalized through an arts-based Indigenist approach. It seeks to develop a culturally safe, reflexive, and practical method for cross-cultural research for the benefit of First Nations Australians.

### Cultural Validation

The above aims and objectives have yet to be culturally validated [25] by the future First Nations co-designers of the study. Therefore, the following details of the study design are also suitably flexible and open to reevaluation [26]. This cultural validation will itself only yield context-specific knowledges that are not necessarily applicable to other First Nations individuals or communities in Australia [27]. This approach and elucidation represents culturally safe research practice that places the locus of power with First Nations peoples to determine whether the specific aims and research processes diminish their cultural identity [28]. Validation will be achieved by triangulating perspectives from culturally appropriate yarning [12] with First Nations community members, non–First Nations researcher ethnographic reflection [29], and through literature review.

### Phase 1: Relational Study

In an endeavor to uphold the Indigenist strategies outlined (data collection), a process of combined autobiography (understanding one’s own life experiences retrospectively and in light of current learning) and ethnography (a process of becoming a participant observer in a culture for the purposes of learning more about others) will be adopted [30]. In this context, this process will be used to produce a rich and accessible body of personal and interpersonal information that can form new directions for further research [31] and is consistent with non–First Nations peoples’ need to reflexively examine themselves and their orientation toward culturally safe practices [32].

### Phase 2: Ritual Co-design

The Jungian nature of the enquiry will guide the co-design of the ritual through Jung’s psychological understanding of spirituality, storytelling, and symbolism. Symbol and metaphor will be used in communication [33,34] using Indigenous methods of yarning [35], dadirri [36], sand talk [13], and photovoice [37].

There is a substantial body of research on First Nations and non–First Nations rituals and ceremonies written from majority Western perspectives [22,38,39]. This research is almost always authored by either international (non-Indigenous) researchers or non–First Nations Australians [40,41]. The academic discussion of ritual (including ritual responsibilities) and ceremony by First Nations Australian voices is in an embryonic but growing phase [42,43]. Female First Nations voices are similarly underrepresented despite the fact that “women played an important role spiritually within Aboriginal society...with their own special ceremonies and stories,” as recounted by Eualeyai and Kamillaroi woman and academic Larissa Behrendt [44]. Evidence for ritual co-design as a method for cross-cultural knowledge creation is absent in landscape research [42,43].

### Ethics Approval

Ethical approval for the phase 1 relational study has been received by the University of New England (UNE) Human Research Ethics Committee (HREC; HE21-142). A separate Human Research Ethics Application [45] for phase 2 of the research project will be submitted to the Aboriginal Health and Medical Research Council (AHMRC)–HREC and the UNE-HREC. Additional ethical protocols will be addressed with the community contacts developed in the phase 1 relational study and will be approached according to the guidelines of the AHMRC-HREC and Australian Institute of Aboriginal and Torres Strait Islander Studies (AIATSIS).

### Consent to Participate

Before any research is undertaken, free, prior, and informed consent will be obtained from the relevant First Nations peoples. The researcher acknowledges that collective consent does not remove the requirement to respect individual rights to participate in Aboriginal and Torres Strait Islander research and that individuals require additional consent [46].

### Participant Recruitment

The development of meaningful relationships is key to genuine and trusting research relationships between First Nations and non–First Nations peoples [47]. The participant recruitment process begins with directly contacting First Nations community members involved at the intersection of landscape research, Country, well-being, and healing. This includes First Nations–controlled community organizations and services and individual community members and leaders [48]. The research team will identify relevant contacts through peer-reviewed literature, personal networks, and searching the internet for similar projects.

The ritual co-design aims to invite 5-10 female collaborators, self-identifying as First Nations Australians, to be a part of this research as cocreators of the design. Female First Nations Australians will be contacted through culturally safe protocols [49], which means developing relationships with community members and leaders and recruiting future co-designers through passive snowball sampling [50].

Permission for participants’ identity to be kept anonymous will be respected, as well as the identity of any family or community members, present or passed, mentioned in the recounting of stories and histories.

### Inclusion and Exclusion Criteria

This research is a gendered enquiry, as informed by successes in similar studies [51-53]. Individuals under 18 years of age will not be included in the study. Due to the inclusive and flexible research philosophy, no individuals are specifically excluded because that will be for First Nations community members to advise according to their local protocols; however, this is a gender-specific Indigenist approach and the lead researcher is a female non–First Nations Australian [54,55].

### Data Collection

For phase 1, the autoethnography and regular critical reflexive practice of the relational study will be conducted simultaneously to, and informed by, social yarning with First Nations community members [50] and in addition to searches of academic journals and grey literature. The researcher (SZ) will undertake weekly reflexive practice through critical reflection [29], which will be converted into Jungian-style mandalas (symbolic diagrams) on a bimonthly basis. No quantitative or qualitative data will be collected from individuals that are consulted during phase 1.

For phase 2, the ritual co-design, data collection methods are underpinned by a value system that prioritizes reciprocal and involved transparent knowledge sharing [35]. Four methods of data collection honoring First Nations Australian methodological praxis will be used: yarning and dadirri [56], storytelling and counter-storytelling [12], sand talk [13], and photovoice [37]. These 4 methods will be used simultaneously to develop a rich, layered, and diverse body of data that will be subject to evaluation within the 3 cycles of the PAR design [57].

Culturally safe landscape research reframes relationships to make research co-designed with First Nations peoples, so their worldviews drive the research to meet their needs [58-60]. This predicts a nonlinear process of data collection more akin to a spider’s web or bricolage of information [61]. Briefly:

1. Yarning is defined by an open dialogue that flows between community members and researchers that lends itself to the development of trust and active participation and accountability on all sides [35]. According to Dr Miriam Rose Ungunmerr-Baumann, a Ngangikurungkurr woman, dadirri (pronounced “da-did-ee”) or “deep listening” refers not only to active listening but also speaks to a willingness to “listen” past the words that are being spoken [56].
2. In psychological research, storytelling and counter-storytelling [33,34] seek to acknowledge power relations and White privilege in research practice. It draws focus to First Nations–led strategies that affords research participants avenues to express personal and collective cosmology, lived experience, and a version of history that challenges that of the dominant society [62].
3. Sand talk is a practical and relational communication method developed by academic Tyson Yunkaporta [13] of the Apalech Clan, of Far North Queensland. This communication technique comprises yarning, dadirri, and storytelling when drawing symbols on sand that articulate complex patterns and concepts.
4. Photovoice is a method of data collection often used in research with women, First Nations, and marginalized communities [63]. Similar studies found that Aboriginal Australian women saw alignment between photovoice methods and cultural customs for sharing knowledge [37]. This research will use photovoice methods to capture and record the symbols of the sand talk yarns.

The interviews, communications pertaining to research cycle planning, yarns, and photovoice discussions will be audio-recorded and transcribed verbatim to assist with data evaluation [64]. Parallels can be drawn between the First Nations methods of data collection—described above—and similar methods used by Jungian and post-Jungian practitioners. For example, sand talk [13] is methodologically similar to sandplay methods developed by post-Jungian academics and practitioners [65,66]. Both sand talk and sandplay use symbol, story, and metaphor to communicate complex emotions and concepts using the medium of sand. Such similarities will be subjected to cultural validation with the future First Nations co-designers of the study before influencing the study design.

### Data Evaluation

The data collected during the ritual co-design will be evaluated based on a three-fold process:

1. The qualitative data generated will be rich, layered, and diverse. As such, the evaluation must be structured to present a cohesive interpretation of the findings [57]. This study will be evaluated through a feminist paradigm, found to be successful with female First Nations community members [51]. However, care will be taken in differentiating feminist theory from First Nations women’s practices, experiences, and ways of knowing [44].
2. This data evaluation will be conducted within the cycles of the PAR design with First Nations co-designers and the wider First Nations community. A key feature of previous successful healing programs [67] has been the First Nations participation in leadership and evaluation of the study design.
3. The qualitative data generated will be coded and evaluated using qualitative text analytics software. Leximancer will be used to identify the most prominent words and themes to produce a map of the key concepts, guiding the researcher to construct a coherent and rigorous evaluation of the rich body of data gathered [68].

### Assessment

Previous researchers cite the importance of practicing critical reflexivity in ensuring cultural safety [69] for First Nations Australians [70,71]. The reflexive data collected during the relational study (autoethnography and reflexivity) will be assessed by First Nations co-designers [26]. The researcher (SZ) will undertake weekly reflexive practice, which will be converted into Jungian-style mandalas. These reflexive writings and mandalas will be yarned about with the First Nations co-designers during the course of the proceeding research cycles. The co-designers themselves will assess whether the reflexive material is evidence of an emerging awareness of cultural safety and respects their cultural identity [28].

The transformation in knowledges of landscape, well-being, and healing will be assessed through combined application of the Environmental Identity scale [26] and the Negative Life Events Scale, which is a measure of emotional and social well-being [72]. The application of such emotional and social well-being scales in First Nations communities in Australia is a sensitive but growing area of research requiring cultural validation [73]. Through an assessment process [25], these scales will be reevaluated, in an effort toward ensuring the cultural safety of the First Nations co-designers [10]. These processes seek to further advance practical evidence in the field of landscape research [60,74].

### Data Management and Sovereignty

In this research, issues of access to data, control of data, data recording, and record keeping are guided by the ethical principles of the AHMRC-HREC [75] and AIATSIS [46]. Ownership, management, and communication of research data and results will be negotiated between First Nations Australians and the researcher at an early stage in the research. This process will also address the lack of evidence of data sovereignty agreements by co-designing an agreement with the future co-designers. The contribution of First Nations Australians’ knowledge, resources, and access to data will also be acknowledged by ensuring open access, enabling First Nations peoples to research results.

### Research Timeline

Taking time to develop trust is an essential first step in developing research with First Nations Australians [76]. In the phase 2: ritual co-design, the 3 research cycles will take approximately 6 months. This timeframe is based on similar collaborative studies that prioritize Indigenist ways of knowing and being [51,77]. This means prioritizing respect regarding commitments and reasonable timeframes, demonstrated by requesting times for meetings that are convenient to community members and holding meetings at community organizations or places nominated by the community members [78]. Therefore, meetings may take longer to schedule and conduct, and hence, the timelines and milestones are suitably flexible.

## Results

The research is in the preliminary stages of participant recruitment for phase 2: ritual co-design. The phase 1 relational study has already been completed. It is expected that data collection for phase 2 will commence in late 2022.

## Discussion

### Expected Findings

It is expected that this qualitative and co-designed project will strengthen cross-cultural co-designer relationships and that the data gathered from these relationships and the accompanying practical outcomes will provide new insight into the interaction between human and landscape well-being. Previous studies have found success when privileging landscape (or Country) when co-designing research with First Nations individuals and communities. This study builds on this evidence base by proposing a novel ritual co-design methodology as a practical method of deepening shared knowledges.

The strength of this study lies in the relational methodology stemming from the Indigenist approach. This approach sees the study prioritizes strong and trusting cross-cultural relationships that form the basis of all research directions and practical outcomes. As a result of these strong relationships, the study is able to respond to community needs, ensuring that outcomes and findings are both meaningful and genuine. A limitation of the study is that the findings will yield context-specific knowledges that are not necessarily applicable to other First Nations or non–First Nations individuals or communities in Australia. However, It is expected that this qualitative data will be able to be used by future researchers to guide directions in qualitative and quantitative research methodology.

### Dissemination Plan

The findings of the research will be continually disseminated throughout the research cycles within the co-designer group and the wider community. This dissemination plan includes attendance at First Nations community meetings as requested; through conducting project information sessions with local community groups; and through peer-reviewed articles, local reports and documents, and conference presentations [50]. This continual dissemination and regular critical reflection is expected to increase rigor in the research through collaborative discussion and subsequent planning of the next step.

## Abbreviations

AHMRC: Aboriginal Health and Medical Research Council
AIATSIS: Australian Institute of Aboriginal and Torres Strait Islander Studies
HREC: Human Research Ethics Committee
PAR: Participatory Action Research
UNE: University of New England

## Data Availability

Ownership, management, and communication of research data and results will be negotiated between First Nations and non–First Nations co-designers and the researcher at an early stage in the research, which will be formalized through a Research Agreement. This process will also address the lack of evidence of data sovereignty agreements by co-designing an agreement. The contribution of First Nations peoples’ knowledge, resources, and access to data will also be acknowledged by ensuring open access, enabling First Nations peoples to access research results.

The data sovereignty agreement will address:

1. Who has ongoing custody of the data
2. Where this data will be stored
3. Who owns the cultural and intellectual property, in particular the data that relates to First Nations Australian knowledges, histories, and traditions
4. How consent for future uses be negotiated
5. How privacy will be maintained if data are used in the future

The raw data of the study will be comprehensively collated into a Microsoft Excel spreadsheet and offered to the future First Nations community to accompany the published reports and documents. Further, upon research completion or closure of the project, the data will be centrally archived and labelled with a persistent identifier to enable future retrieval. The metadata (describing the research data) of the research data and/or materials will be recorded in the Metadata Store of the Library Services of the University of New English (UNE). Once the metadata record has been completed, the record will be issued with a digital object identifier and made publicly accessible in Research UNE (RUNE) and Research Data Australia. This procedure reflects the UNE’s open access policies and procedures, which encourage researchers to share and publish data and metadata records.
